# Supplementary material for: Olfactory Neuromodulation of Motion Vision Circuitry in Drosophila
Source: Curr Biol. 2015 Feb 16;25(4):467–72. doi: 10.1016/j.cub.2014.12.012 (PMC4331282; doi:10.1016/j.cub.2014.12.012)
Supplement: Document S1. Supplemental Experimental Procedures [file mmc1.pdf]

Current Biology
Supplemental Information

Olfactory Neuromodulation of Motion Vision Circuitry in *Drosophila*

Sara M. Wasserman, Jacob W. Aptekar, Patrick Lu, Jade Nguyen, Austin L. Wang, Mehmet F. Keles, Anna Grygoruk, David E. Krantz, Camilla Larsen, and Mark A. Frye

Supplemental Experimental Procedures

Behavior

Closed Loop Magnetic Tether Flight Simulator

A 30 deg wide vertical stripe was rotated around the arena for 60 s at the start of each experiment to verify that an individual animal was able to appropriately orient at all points around the arena. This same stripe was oscillated at 90 deg to visually attract the flies to the odor nozzle. A static wide-field pattern (30 deg spatial wavelength, 94% pattern contrast, 78 cd/ m^2) was presented for the duration of each experiment. Odor was delivered at the location marked as 180-deg and odor stimuli included apple cider vinegar (Ralphs Brand) or water. Individual flies were not run more than three times through a single experiment and animals that stopped flying more than three times were discarded. Flies included in the analyses were required to have acquired the odor plume for at least one frame (30 frames/sec) within the first five seconds. Analyses were performed using custom-written MATLAB software as previously described [S4,S5].

Rigid Tether Flight Simulator

Each experiment began with a period of closed-loop bar fixation wherein the fly is able to actively control the position of the bar[S6]. Flies that were not able to stabilize the bar at the start of an experiment were discarded. Flies were presented with 8 seconds of open-loop ground motion from one of 8 randomly selected spatial stimulus patterns. Half of the trials were randomly paired with an apple cider vinegar plume and half with a water plume. Each test trial was interspersed with periods of closed-loop bar tracking.

Calcium Imaging

Once anesthetized, flies were wedged into a 1/32 inch slit cut into 0.001 inch thick stainless steel shim (304 Stainless Shim. Trinity Brand Industries. Countryside, IL) in a custom-built acetal stage. The cervical connective was flexed downwards to bring the posterior surface of the head capsule flush with the shim, and the rim of the cuticle was fixed in place with dental acrylic[S7]. The proboscis was fixed to prevent motion. The posterior cuticle was cut out and removed with a pair of sharpened Dumont #5 forceps (Fine Science Tools. Foster City, CA). Overlying tissues were removed to provide clear access to the optic lobe. During imaging, a perfusing solution was flowed through the well at a rate of ~1ml/min via a gravity drip regulated by an in-line valve. Perfusate passed through an in-line temperature regulator (Warner Instruments. Hamden, CT) at 19 degrees C, resulting in well temperature of 21-23 degrees C throughout the experiment. Perfusate saline is based on [S8].

Visual Stimuli for Odd tuning experiments

To characterize the subset of the ODD cell group with dendrites in the lobula plate, we performed both directional and spatiotemporal frequency-tuning experiments. In each case, we used square-wave gratings at 100% contrast. For the directional tuning, animals were shown a square-wave grating with a 27 deg spatial wavelength that drifted at a velocity of 27

deg/s for 5 seconds. For each trial, the grating was oriented along one of 36 evenly spaced directions spanning the circle. Each experiment consisted of a single repetition of these 36 trials, presented in random order, interleaved with 2 second rest periods. For spatiotemporal frequency tuning, animals were presented with a square wave grating with a spatial period of 27 deg was drifted in the preferred direction (back-to-front) at temporal frequencies indicated in Figure 2H.

Image Processing

Acquired movies were aligned in two dimensions to a reference image (typically the first frame of the movie) with a built-in two-dimensional correlation algorithm (Slidebook, Intelligent Imaging Innovations, Inc. Denver, CO). An image of the mean fluorescence intensity over time was then exported to MatLab where a customized algorithm was used to parcelate the image into isoluminant regions of interest (ROIs). All data was downsampled to 10Hz. $\Delta F/F$ values were calculated by dividing by the mean fluorescence over frames 30 to 70 (3-7s). Mean maximum $\Delta F/F$ values were identified within 20 frames on either side of the maximum response during the stimulus period.

HSE , Hx and T4T5 imaging

A single identifiable ROI was selected for Hx and HSE imaging with GCaMP6m. [S9, S10] ROIs for T4T5 were manually assigned to three groups according to the dendritic or axonal locations within the medulla, lobula, or lobula plate. Responses from all three regions did not show significant difference from each other. Therefore, we display only responses within the lobula plate as a representative (Figure 3). Tdc2-Gal4 processing utilized GCaMP6s [S9].

Semi-automatic noise removal for T4T5 and Tdc2

Unlike the tangential cells Hx and HSE, T4T5 and Tdc2 cells are densely tiled within the lobula plate, thus it was necessary to measure their response to stimuli as a population, rather than from a single, repeatable ROI. We began by tiling the movie-recording with a mask that subdivided each recording into equiluminant channels. To remove background ROIs and noise, we developed a recursive principal component analysis approach. To avoid privileging bright ROIs in our analysis, we subtracted the mean value from each row of the matrix and normalized by the standard deviation of luminance over the movie. We then treated each sample as an independent component and each ROI as an independent measurement of that component. We next performed Principle Components Analysis (PCA) and used k-means clustering to group ROIs based off similarity of coefficients across the first four principal components. We identified outlying clusters of points and excluded or included these clusters from further analysis if the non-normalized, non-mean subtracted was low. These ROIs generally corresponded to the background or non-responsive parts of the image. ROIs that responded in a systematically different manner from another subset were separated for the next level of analysis. Once we excluded background ROIs from further analysis, we then performed the same analysis on the remaining ROIs and again looked for systematic differences by visualizing the projections of the remaining ROIs into a new space of principal components. Once we no longer found systematic sources of variation, the process

terminated. As reported in this study, this technique requires user interaction, although it could be automated.

Tdc2 ROI Pruning

Because the Tdc2 cellular processes are diffuse and small in caliber within the lobula plate, we found these recordings to be highly sensitive to animal motion. For this reason, before performing the recursive principle components analysis to remove noise pixels and background, we first excluded all animals where the recordings showed a large amount of motion artifacts that could not be resolved by automatic image alignment. The reported results are from N=6 animals.

Confocal Imaging

Images shown in Figures 2B, 3D, 3G, 4C were obtained via the following protocol. 2-5 days old female flies were dissected in 1x PBS and fixed in 4% paraformaldehyde followed by 3X15 minutes washes in 1x PBS/ 0.3% Triton X-100 (PBST). Brains were then blocked in 5% goat serum diluted in 0.3% PBST for 30 min and incubated in primary antibodies 2 days at (4 °C). Following 3x15 minutes washes in PBST, brains were incubated in secondary antibodies 2 days at (4 °C). Lastly, brains were mounted in Vectashield (Vector Laboratories) on a microscope slide. 2 µm stacks were taken using a Zeiss 710 confocal microscope and images were analyzed with ImageJ (National Institutes of Health). The following primary and secondary antibodies were used: mouse anti-nc82 (1:10, Developmental Studies Hybridoma Bank, University of Iowa, http://dshb.biology.uiowa.edu/bruchpilot), rabbit anti-GFP (1:1000, Molecular Probes, A11122), goat anti-rabbit Alexa 488 (1:200, Molecular Probes, A11034) and goat anti-mouse Alexa 568 (1:200, Molecular Probes, A11031)

GFP reconstitution across synaptic partners (GRASP)

Adult brains were dissected in cold PBS and fixed in a 4% paraformaldehyde/PBS solution for 30 min at room temperature. Brains were then washed several times in PBS containing 0.5% Triton X-100 (PBT) followed by a 2 hour incubation in 1% goat serum (GS) before antigen detection with primary antibody overnight at 4 C° in PBT + 0.5% GS. Following primary antibody incubation brains were washed for 8 hours and incubated overnight in secondary antibody at 4 C° followed by washing for 1 day. Brains were mounted in PBS and viewed using a Zeiss 510 confocal microscope using an air x20. The following antibodies were used: mouse monoclonal anti-Bruchpilot (nc82) (Developmental Studies Hybridoma Bank) (1:10 dilution) and polyclonal rabbit anti-GFP (Invitrogen) (1:400 dilution). Secondary antibodies (Invitrogen) were: Alexa Fluor 488 donkey anti-rabbit, and Alexa Fluor 546 goat-anti-mouse. These were used at a 1:500 dilution.

Supplemental references

S1. Levy, P., and Larsen, C. (2013). Odd-skipped labels a group of distinct neurons associated with the mushroom body and optic lobe in the adult Drosophila brain. J. Comp. Neurol. *521*, 3716–3740.

S2. Burke, C. J., Huetteroth, W., Owald, D., Perisse, E., Krashes, M. J., Das, G., Gohl, D., Silies, M., Certel, S., and Waddell, S. (2012). Layered reward signalling through octopamine and dopamine in Drosophila. Nature *492*, 433–437.

S3. Gordon, M. D., and Scott, K. (2009). Motor control in a Drosophila taste circuit. Neuron *61*, 373–384.

S4. Duistermars, B. J., and Frye, M. A. (2008). Crossmodal visual input for odor tracking during fly flight. Curr. Biol. *18*, 270–275.

S5. Maimon, G., Straw, A. D., and Dickinson, M. H. (2008). A simple vision-based algorithm for decision making in flying Drosophila. Curr. Biol. *18*, 464–470.

S6. Chow, D. M., Theobald, J. C., and Frye, M. A. (2011). An olfactory circuit increases the fidelity of visual behavior. J. Neurosci. *31*, 15035–15047.

S7. Seelig, J. D., Chiappe, M. E., Lott, G. K., Dutta, A., Osborne, J. E., Reiser, M. B., and Jayaraman, V. (2010). Two-photon calcium imaging from head-fixed Drosophila during optomotor walking behavior. Nat. Methods *7*, 535–540.

S8. Wilson, R. I., Turner, G. C., and Laurent, G. (2004). Transformation of olfactory representations in the Drosophila antennal lobe. Science *303*, 366–370.

S9. Chen, T.-W., Wardill, T. J., Sun, Y., Pulver, S. R., Renninger, S. L., Baohan, A., Schreiter, E. R., Kerr, R. a, Orger, M. B., Jayaraman, V., et al. (2013). Ultrasensitive fluorescent proteins for imaging neuronal activity. Nature *499*, 295–300.

S10. Akerboom, J., Chen, T.-W., Wardill, T. J., Tian, L., Marvin, J. S., Mutlu, S., Calderon, N. C., Esposti, F., Borghuis, B. G., Sun, X. R., et al. (2012). Optimization of a GCaMP calcium indicator for neural activity imaging. J. Neurosci. *32*, 13819–13840.
