# Supplementary material for: Olfactory Neuromodulation of Motion Vision Circuitry in Drosophila
Source: Curr Biol. 2015 Feb 16;25(4):467–72. doi: 10.1016/j.cub.2014.12.012 (PMC4331282; doi:10.1016/j.cub.2014.12.012)
Supplement: Document S2. Article plus Supplemental Information [file mmc2.pdf]

Report

Current Biology

Olfactory Neuromodulation of Motion Vision Circuitry in *Drosophila*

Highlights

- Small-field motion detection neurons are required for odor-tracking behavior
- Responses of a directional wide-field interneuron (Hx) increase with paired odor
- Odor activates octopaminergic (OA) neurons that innervate the visual system
- OA cells contact Hx; OA vesicle trafficking is required for odor-tracking behavior

Authors

Sara M. Wasserman,
Jacob W. Aptekar, ..., Camilla Larsen,
Mark A. Frye

Correspondence

frye@ucla.edu

In Brief

Wasserman et al. report that a directionally selective wide-field motion-detecting neuron (Hx) in the fly increases response gain with paired odor. This multimodal interaction is dependent upon vesicle trafficking from octopaminergic neurons, which are themselves responsive to odor and make cell-cell contact with Hx.

Wasserman et al., 2015, Current Biology *25*, 467–472
February 16, 2015 ©2015 The Authors.
http://dx.doi.org/10.1016/j.cub.2014.12.012

Current Biology 25, 467–472, February 16, 2015 ©2015 The Authors http://dx.doi.org/10.1016/j.cub.2014.12.012

Report

Olfactory Neuromodulation of Motion Vision Circuitry in *Drosophila*

Sara M. Wasserman,[1,4] Jacob W. Aptekar,[1,4] Patrick Lu,[1] Jade Nguyen,[1] Austin L. Wang,[1] Mehmet F. Keles,[1] Anna Grygoruk,[2] David E. Krantz,[2] Camilla Larsen,[3] and Mark A. Frye[1,*]
[1]Howard Hughes Medical Institute and Department of Integrative Biology and Physiology, University of California, Los Angeles, Los Angeles, CA 90095, USA
[2]Department of Psychiatry and Biobehavioral Sciences, David Geffen School of Medicine, University of California, Los Angeles, Los Angeles, CA 90095, USA
[3]Medical Research Council Centre for Developmental Biology, King's College London, London SE1 1UL, UK

Summary

It is well established that perception is largely multisensory [1]; often served by modalities such as touch, vision, and hearing that detect stimuli emanating from a common point in space [2, 3]; and processed by brain tissue maps that are spatially aligned [4]. However, the neural interactions among modalities that share no spatial stimulus domain yet are essential for robust perception within noisy environments remain uncharacterized. *Drosophila melanogaster* makes its living navigating food odor plumes. Odor acts to increase the strength of gaze-stabilizing optomotor reflexes [5] to keep the animal aligned within an invisible plume, facilitating odor localization in free flight [6–8]. Here, we investigate the cellular mechanism for cross-modal behavioral interactions. We characterize a wide-field motion-selective interneuron of the lobula plate that shares anatomical and physiological similarities with the "Hx" neuron identified in larger flies [9, 10]. *Drosophila* Hx exhibits cross-modal enhancement of visual responses by paired odor, and presynaptic inputs to the lobula plate are required for behavioral odor tracking but are not themselves the target of odor modulation, nor is the neighboring wide-field "HSE" neuron [11]. Octopaminergic neurons mediating increased visual responses upon flight initiation [12] also show odor-evoked calcium modulations and form connections with Hx dendrites. Finally, restoring synaptic vesicle trafficking within the octopaminergic neurons of animals carrying a null mutation for all aminergic signaling [13] is sufficient to restore odor-tracking behavior. These results are the first to demonstrate cellular mechanisms underlying visual-olfactory integration required for odor localization in fruit flies, which may be representative of adaptive multisensory interactions across taxa.

Results and Discussion

In addition to feedback from head movements [14–18], a fly in flight stabilizes its gaze by optomotor steering movements of the wings that turn the whole body [19]. The strength of steering optomotor responses increases when flies experience an appetitive odor [5]. Here we tethered a fly rigidly within a flight simulator composed of a wrap-around electronic display and equipped with an odor port (Figure 1A) to measure the optomotor impulse response to a rapid rotation of the visual panorama [21]. Pairing an appetitive food odor (vinegar) with the visual stimulus results in a roughly 40% increase in the optomotor response (OMR), which is assessed by measuring the mean difference in wing beat amplitude across the two wings (ΔWBA) elicited by an impulse in yaw velocity (Figure 1B), consistent with prior measurements [5].

Optomotor responses in *Drosophila* can be elicited by optogenetic activation of tangential wide-field collating neurons HSE and HSN housed in the third optic ganglion, the lobula plate [22]. To examine whether motion integrating circuitry of the lobula plate is involved in odor-enhanced OMRs, we genetically hyperpolarized the small-field columnar neurons T4 and T5, which supply retinotopic motion signals to the lobula plate [23]. Using the same magnetic-tether flight simulator (Figure 1C) applied to demonstrate the dependence of self-generated visual motion signals for active plume tracking [24], we measured the animals' ability to locate and stabilize their heading within a vinegar plume. We divided plume-tracking behavior into three components: (1) initial detection, defined by the proportion of flies that oriented themselves within ±10° of the odor nozzle—flies that did not do so were not included in the subsequent analysis; (2) acquisition, defined by time spent within the plume over the first 10 s of the trial; and (3) continuous tracking, defined by how much of the final 10 s of the trial the fly spent oriented within the plume (Figure 1D). We found no significant difference between the proportions of T4T5-blocked versus control flies that detected the plume (chi-square test, $p > 0.05$). Similarly, blocking T4T5 did not significantly alter the mean time spent in the plume during the acquisition phase, but T4T5-blocked flies were unable to sustain plume tracking for the duration of the trial, in comparison to controls (Figures 1D and 1E). This shows that whereas the lack of motion signals carried by T4T5 to the lobula plate does not compromise the animals' ability to detect or initially localize an odor plume, local motion signals are required to stabilize flight heading within the plume. This is consistent with the finding that switching the high-contrast grating displayed in the flight arena to an equiluminant grayscale, thereby reducing optic flow generated by the fly's own movements, eliminates its ability to remain within the plume [24].

A lobula plate tangential cell (LPTC) was recently identified anatomically in *Drosophila*, along with a number of neurons within higher-order olfactory regions of the mushroom bodies, by its shared expression of the *Odd-skipped* transcription factor [9]. The tangential dendritic arbor of this LPTC spans the dorsal projection of the lobula plate (Figure 2Bi), tightly restricted to layer 2 (Figures 2Bii and 2Biii), which is the layer receiving back-to-front directional motion input from the columnar T4T5 terminals [23]. The axon projects heterolateral to the cell body and dendrites [9]. To characterize its motion-coding properties, we expressed a genetically encoded calcium indicator, GCaMP6m [25], under the Odd-Gal4 driver [9] and recorded cellular activity under a two-photon excitation

[4]Co-first author
*Correspondence: frye@ucla.edu
This is an open access article under the CC BY license (http://creativecommons.org/licenses/by/4.0/).

CrossMark

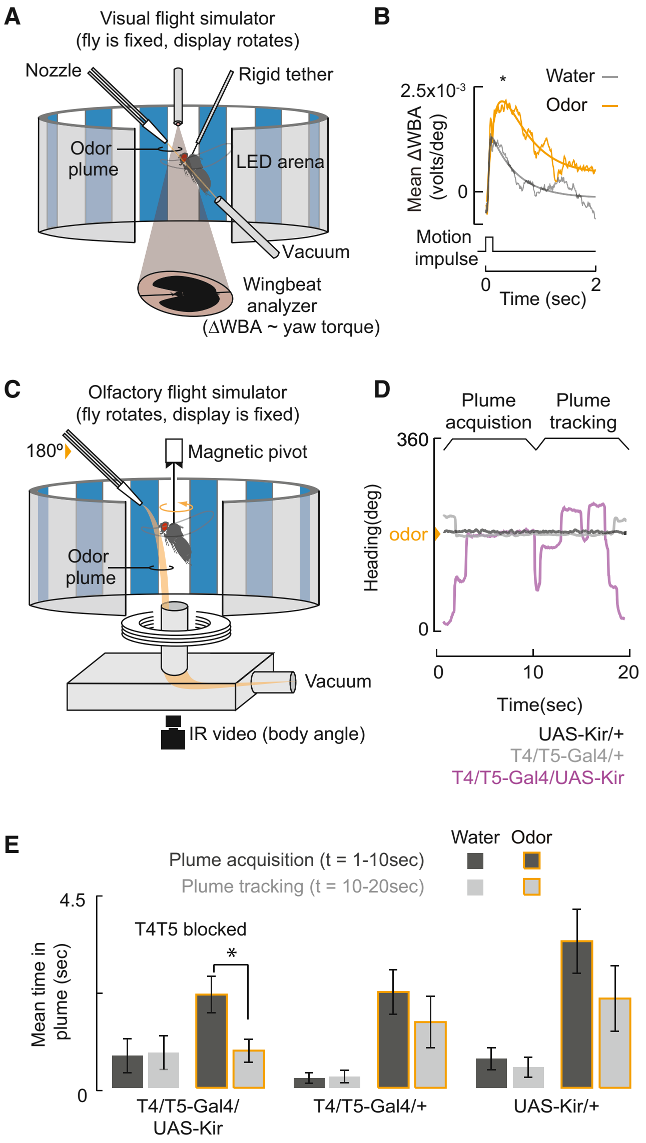

Figure 1. Visual and Olfactory Information Are Integrated to Generate More Robust Behavioral Outputs

(A) The electronic visual flight simulator records wing kinematics from a fixed fly in response to sensory stimuli. The difference in wing beat amplitude (ΔWBA) across the two wings is proportional to yaw torque. Steering torque is activated by movement of the panoramic grating projected on the circular display of light-emitting diodes (LEDs) [20]. The arena is equipped with a laminar flow olfactometer.

(B) Average modulation of ΔWBA optomotor response to a velocity impulse in the yaw axis with and without paired odor presentation. The sum of two exponential functions is fitted to the impulse responses (smooth line). Asterisk indicates two-way paired t test, $p < 0.05$ comparing peak amplitude values of fits to responses by individual flies. n = 15.

(C) Magnetic-tether flight simulator records body orientation in response to a spatially restricted odor plume. A video image tracks the fly's angular heading changes on a magnetic tether allowing free movement in the yaw plane. A narrow plume of odor is delivered from one side of the arena.

(D) Exemplar flight orientation responses to an odor plume located at 180° (as in C) shown for T4T5-blocked flies (purple trace) and parental controls (black and gray traces).

(E) Inactivation of the T4T5 local motion-detecting neurons (T4T5-Gal4/UAS-Kir, n = 25) inhibits stabilization of odor plume tracking. Time in plume, for each category acquisition and tracking, is total time spent within ±10° of the odor nozzle over the time period defined in (D). T4T5-Gal4/+, n = 20; UAS-Kir/+, n = 19). Mean ± SEM are shown. Asterisk denotes significant difference (two-way paired t test, $p < 0.05$).

imaging system equipped with an LED display [26] (Figure 2A). Imaging from dendritic regions of interest (ROIs) (see Experimental Procedures) in response to a narrow vertical bar, we demonstrate that this cell is excited by back-to-front motion across the ipsilateral eye within a 50° receptive field positioned just ipsilateral to the visual midline (Figure 2C) and is more excited by progressively wider randomly textured bars (Figure 2D). We found no systematic response differences within small ROIs spanning the tangential dendritic arbor (data not shown) and therefore focused subsequent imaging analysis on a primary dendritic branch that was identifiable in each preparation (Figure 2B, white box). To further explore wide-field response properties, we varied the orientation of a full-field grating, demonstrating that this cell is strongly tuned to front-to-back motion oriented along the horizontal body axis (Figure 2E) and, like other wide-field *Drosophila* LPTCs [11, 23], exhibits a 1 Hz temporal frequency optimum (Figure 2F). The matched directional preferences and layer specificity strongly suggest that Hx receives local motion signals from the T4T5 system but do not preclude other potential inputs. The neuronal morphology and receptive-field properties of this cell are strongly reminiscent of the Hx neuron characterized in blowflies [10], and we refer to it thusly hereafter.

Motivated by the transcription-factor spatial profile shared with higher-order olfactory projection neurons, we sought to determine whether Hx was cross-modally activated by odor. The two-photon recording preparation and LED display was equipped with a laminar flow olfactometer (Figure 2A). We presented a regime of five repeated 10-s epochs of back-to-front wide-field motion interspersed with rest periods. The second motion epoch was accompanied by a 10-s odor pulse (delivered bilaterally). There was a subtle yet significant increase in the motion-elicited excitatory response of Hx during paired odor presentation (epoch 2, Figure 3A), observed within each individual fly preparation tested (Figure 3B) but absent in water vapor controls (Figure 3C). To determine whether the primary site of visual-olfactory integration resided with Hx or the local motion detectors presynaptic to the lobula plate, we performed the same experiment and recorded the intracellular activity of T4T5 cells. The T4T5-Gal4 driver labels cell processes within the medulla, lobula, and lobula plate ([22, 23] and Figure 3D), and we found no differences between responses from ROIs imaged within the processes of these neuropils (chi-square test, $p > 0.05$), nor did we observe any changes in the excitatory motion responses of T4T5 ROIs found within the lobula plate upon paired odor presentation (Figures 3E and 3F). These results reject the possibility that odor-enhanced responses in Hx represent general arousal phenomena and confirm that the site of cross-modal interaction resides within wide-field-integrating lobula plate neurons rather than presynaptic local motion detectors. To assess whether odor activates all LPTCs, we examined the activity of HSE, a neighboring neuron to Hx that is selective for horizontal motion (HSE [11]; Figure 3G), but we did not observe odor-evoked changes in the visual responses of this cell (Figures 3H and 3I).

Visual responses by LPTCs are modulated by the onset of locomotion [27, 28], and this increase in response gain is mediated by octopaminergic innervation [29–31]. We reasoned that octopamine release might also be triggered by olfactory signaling within the visual system to modulate Hx responses.

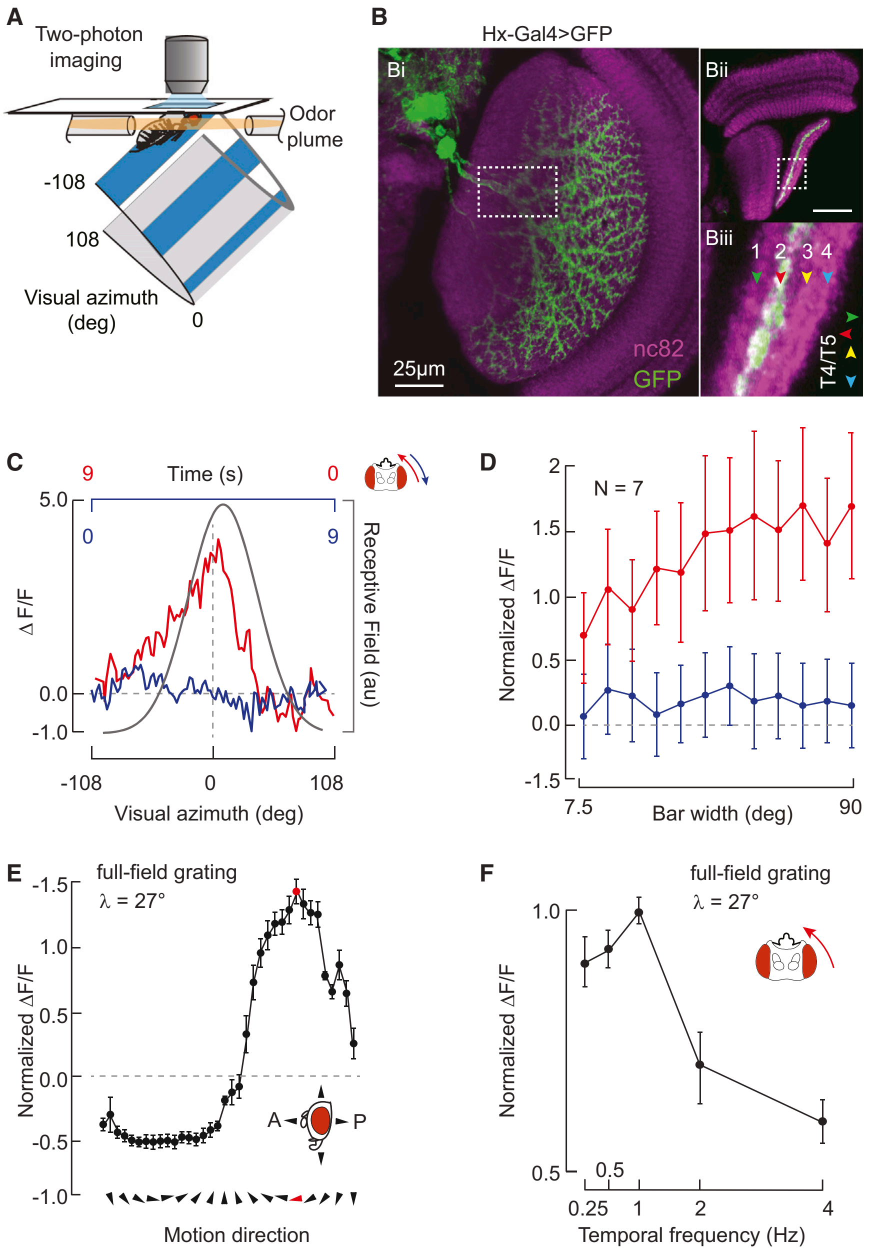

Figure 2. Two-Photon Calcium Imaging and Characterization of Hx Tuning Properties

(A) Perspective-matched LED arena display within the imaging apparatus, equipped with an olfactometer.

(Bi) Posterior view confocal image of Hx dendrites within the right lobula plate via Odd-skipped-Gal4/GFP. The dashed white rectangle indicates the imaging ROI. Neuropil is indicated in purple (nc82 staining).

(Bii) Dorsal view of Hx dendrites within the lobula plate via Odd-skipped-Gal4/GFP (same as Bi). White dashed box within the lobula plate indicates enlarged region shown in (Biii). Scale bar, 25μm.

(Biii) Enlarged cross-section of lobula plate demonstrates the four layers of the lobula plate, with Hx innervation restricted to layer 2. Arrowheads indicate layer-specific directional tuning of T4T5 innervation [23].

(C) Average ΔF/F from Hx in response to a vertical bar revolving in each of two horizontal directions, either back to front (red) or front to back (blue), across the full 216° display. Black line represents superimposed ipsilateral azimuthal receptive-field fit. n = 7 animals.

(D) Mean responses ± SEM to a vertical bar of varying width revolving in each of two horizontal directions across the display. n = 7 animals.

(E) Directional tuning of Hx. A square-wave grating (27° wavelength) was moved in each direction as indicated on the x axis, and maximum ΔF/F was normalized to the largest response observed. Points indicate mean responses ± SEM. Red point and arrowhead indicate the stimulus direction giving maximum response, used in (F). n = 7 animals.

(F) Temporal frequency tuning of Hx. A square-wave grating was moved at constant velocity from back to front. Points indicate mean responses ± SEM. n = 7 animals.

We first determined that the octopaminergic terminals innervating the lobula plate show increased GCaMP fluorescence in response to an odor pulse (Figure 4A), which was demonstrated in each fly tested (Figure 4B). To examine whether these octopaminergic interneurons make synapses with Hx, we made use of a genetic construct that recombines GFP between two cells in close contact (GFP reconstitution across synaptic partners [GRASP] [32]). Expressing one inactive half of the split-GFP within the Tdc2 octopaminergic neurons and the other half within Hx resulted in GFP puncta distributed within the lobula plate (Figure 4C) in a pattern similar to the dendritic profile of Hx (Figure 4C, inset), indicating synapses or other close cell-cell connections such as gap junctions between Tdc2 and Hx. In addition to implicating Tdc2 in the olfactory modulation of Hx, our GRASP data also support prior findings demonstrating that octopaminergic signaling in the brain is necessary for locomotion-induced gain in LPTCs [12].

Likely owing to the role of *Odd-skipped* in development, driving neuronal inactivators with Hx-Gal4 is lethal and nevertheless would have been impossible to evaluate for visual-olfactory integration due to its expression in both visual and olfactory centers [25]. Therefore, we reasoned that if octopaminergic modulation of visual circuitry is important for odor-tracking behavior, then the absence of octopaminergic signaling throughout the brain should strongly perturb odor-tracking behavior. To test this hypothesis, we used a fly strain carrying a null (loss-of-function) mutation in the *Drosophila* vesicular monoamine transporter (*dVMAT*) [13]. Rescue with a DVMAT transgene in octopaminergic neurons, but not with dopaminergic or serotonergic neurons, is sufficient to restore plume-tracking behavior (Figure 4D). As a negative control, we tested animals rescued with a DVMAT trafficking mutant (Tdc2-Gal4/Δ3VMAT [13]); these animals were unable to maintain their heading within the odor plume of the olfactory flight simulator (Figure 4D). These three lines of evidence—odor activation of Tdc2 cells, GFP puncta (GRASP) between Hx and Tdc2 neurons, and the rescue of olfactory tracking when synaptic release by octopaminergic cells is restored—provide a parsimonious interpretation that

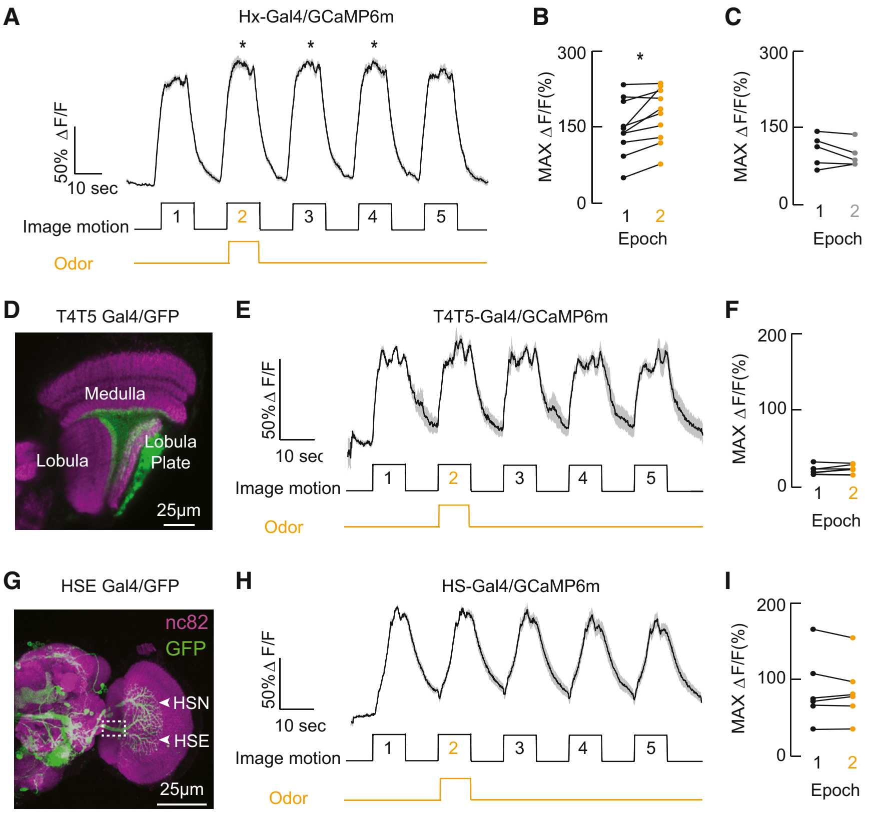

Figure 3. Odor-Induced Modulation of Hx Activity

(A) Intracellular calcium response to visual motion by Hx neurons expressing GCaMP6m. Mean ΔF/F ± 1 SEM is shown. *p < 0.05, rank-sum test on peak response amplitude compared between epoch one (prior to odor stimulation) and each following epoch, n = 13 animals.

(B) Mean maximum ΔF/F from each individual fly compared between epochs one (prior to odor stimulation, black circle) and two (paired odor, orange circle) from (A). *p < 0.05, rank-sum test, n = 13 animals.

(C) Mean maximum ΔF/F from each individual fly compared between epochs one (prior to odor stimulation, black circle) and two (water vapor control, gray circle) from (A). Epochs one and two were not statistically different via rank-sum test, n = 6 animals.

(D) T4T5-Gal4 expression pattern within the visual ganglia. ROIs shown in (E) are from the lobula plate.

(E) Mean ΔF/F ± 1 SEM for T4T5 terminals in the lobula plate. n = 6 animals.

(F) Mean maximum ΔF/F for T4T5 terminals for individual animal in epochs one and two. n = 6 animals.

(G) R27B03-Gal4 expression pattern includes HSE neurons within the lobula plate, imaged within the primary HSE dendrite (white dashed box).

(H) Mean ΔF/F ± 1 SEM for HSE. n = 7 animals.

(I) Mean maximum ΔF/F for HSE for each individual animal in epochs one and two. n = 6 animals.

odor-driven octopamine release modulates the gain of visual circuitry.

Octopamine mediates locomotion-induced modulation of another LPTC, the HSE neuron [12], which is not activated by odor (Figure 3H). This provides an exciting experimental platform for broader investigation into how aminergic signaling differentially modulates postsynaptic targets within the same neuropil. It is possible that, like Hx, HSE is also modulated by odor, but that the effect is observable only when superposed with a flight-activated increase in visual response gain [33]. Additionally, like norepinephrine, octopamine acts through multiple receptor-signaling pathways having wide-ranging influences over cellular physiology. One receptor class (OCTα-R) increases calcium entry, while another (OCTβ-R) elevates intracellular cAMP levels [34] to act as either an agonist or an antagonist on synaptic and behavioral plasticity in an octopamine receptor-dependent fashion [34]. Differential receptor expression could in turn mediate differential octopaminergic neuromodulation of visual circuitry.

In summary, we have revealed a novel cellular cross-modal interaction that could support behavioral findings whereby food odor detection increases visual stability in an odor plume. Future work could elaborate additional neuronal pathways supporting related cross-modal behaviors such as enhanced salience of visual objects by odor [35]. These cross-modal interactions provide a mechanism to dynamically enhance sensory perception in a contextually appropriate manner.

Experimental Procedures

Animals

For behavior experiments, we used wild-type *D. melanogaster*, 3- to 6-day-old posteclosion females. Other lines used for behavior and imaging experiments included T4T5-Gal4 (Bloomington ID 40034), Tdc2-Gal4 (Bloomington ID 9313), UAS-Kir2.1-EGFP (Bloomington ID 6596), UAS-mCD8::GFP (Bloomington ID 5137), HSE-Gal4 (Bloomington ID 49211), UAS-GCaMP6m (Bloomington ID 42748), UAS-GCaMP6s (Bloomington ID 42749), and Odd-Skipped-Gal4 [9]. GRASP constructs were generated using the transgenes *Odd-Gal4* [9], *Tdc2-LexA* [36], and *UAS-CD4::spGFP1-10; LexAop-CD4::spGFP11* [37]. Random individuals were selected from a population for each experimental group according to genotype. No experimenter blinding was done.

Behavior

Closed-Loop Magnetic-Tether Flight Simulator

The magnetic-tether flight arena allows a fly to steer freely in the yaw plane, allowing assessment of odor plume-tracking capability, and has been described in detail previously [6, 24, 38].

Rigid-Tether Flight Simulator

The rigid-tether arena records a fixed fly's wing kinematic responses to visual stimuli, closing a feedback loop to allow the animal to control the velocity of image motion on the display or allow the assessment of visual response gain under open-loop feedback conditions, and has been described in detail previously [5, 20]. Odor was delivered through a narrow nozzle as reported previously [5].

In order to quantify the response of the fly to panoramic yaw motion, we use a white-noise method for estimating the yaw impulse response for each individual animal. The impulse response, *g(t)*, of the fly's steering plant is measured by cross-correlating a time-varying and spectrally broad sequence of velocity impulses, *x(t)*, with the time-varying output signal produced by difference in wing beat amplitudes (ΔWBA), *y(t)*. The kernel function [5] represents the steering response to an impulsive step in the pattern display position by one pixel (3.75°). Impulse responses to water control and odor [5] were fit and parameters were calculated as described previously [39].

Calcium Imaging

Adult female *D. melanogaster* expressing the genetically encoded calcium indicator GCamp6m [40] under one of the four Gal4 drivers were anesthetized under cold sedation. Imaging was performed with a two-photon

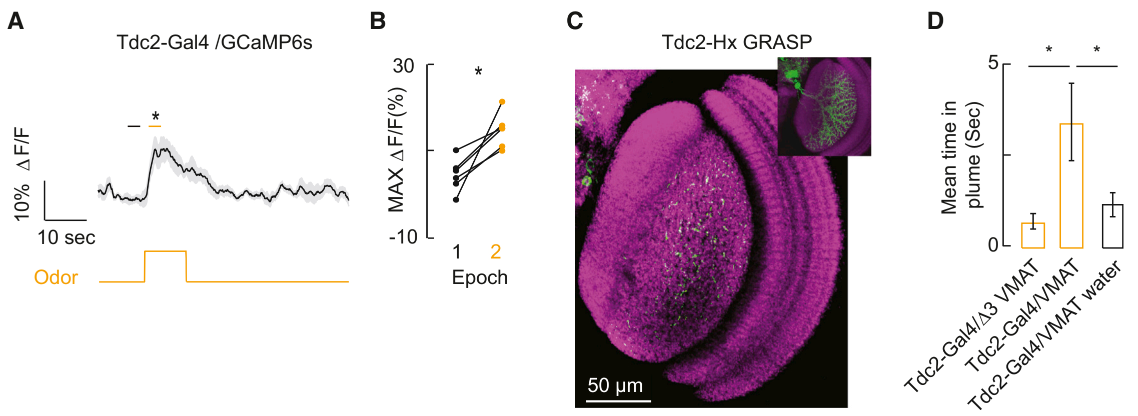

Figure 4. Octopaminergic Neurons Innervating the Lobula Plate Are Activated by Odor, Make Close Contact with Hx, and Are Required for Behavioral Plume Tracking

(A) Intracellular calcium dynamics (ΔF/F, GCaMP6s) of octopaminergic terminals innervating the lobula plate in response to olfactory stimulation. Asterisks indicates significance between odor off (black line) and odor on (orange line) shown above the mean ΔF/F response (two-way paired t test, $p < 0.005$). n = 6 animals.

(B) Mean maximum ΔF/F for each individual animal during a period preceding the odor pulse (black) and during the odor pulse (orange). Horizontal bars over the ΔF/F response in (A) indicate the measurement epochs. n = 6 animals.

(C) GFP expression by GRASP indicates octopaminergic (Tdc2-Gal4) connections with Hx (Odd-Gal4). Inset shows Hx arborization pattern to highlight similarity in GFP profile between GRASP and the lobula plate tangential cell.

(D) Mean time ± SEM spent in odor plume during the duration of the experiment (olfactory flight simulator; Figure 1C) for flies carrying a null mutation in the *Drosophila* vesicular monoamine transporter *dVMAT* rescued with either a wild-type DVMAT transgene in octopaminergic neurons (Tdc2-Gal4/ VMAT) or a DVMAT trafficking mutant (Tdc2-Gal4/ Δ3VMAT). Asterisk indicates significant difference (two-way paired t test, $p < 0.05$) between VMAT (n = 32 animals) and Δ3VMAT (n = 21 animals). Also shown is mean time in plume for Tdc2-Gal4/VMAT-rescued flies exposed to water rather than vinegar (n = 32 animals, *$p < 0.05$ by two-way paired t test).

excitation scanning microscope (Intelligent Imaging Innovations). We used a 20×/NA 1.0 water-immersion objective lens (Carl Zeiss). Laser power was regulated to 10–20 mW measured at the focus of the objective lens. Images were collected at 8–11 Hz and 300–500 nm/pixel. Temporal registration with input stimuli was achieved by recording a voltage pulse at the completion of each frame that was output to our data acquisition device (National Instruments). Visual stimulus was produced by a 12–20 panel arena that was oriented orthogonal to the anterior-posterior axis of the head, subtending 216° of visual azimuth and 120° of elevation on the retina (IO Rodeo) using open-source MATLAB packages (https://bitbucket.org/mreiser/panels/src). Stimulus and data acquisition were controlled by custom-written software in MATLAB (The Mathworks).

Visual Stimuli for Sensory Integration Experiments

One of eight randomly textured display patterns was selected at random for each trial and held static for 10-s periods of rest or 10-s periods of motion stimulation. For odor recordings from Tdc2-Gal4 neurons, the visual pattern was on but stationary. In all visual motion experiments, the pattern moved with a velocity of 22°/s for 10 s. Preparations that showed too much movement artifact or fluorescent bleaching, or that did not demonstrate ΔF/F responses over background levels for at least two presentations of the full stimulus set, were excluded from analysis.

Odor Delivery

Odor was injected at 50 ml/min (Sensirion mass flow controller) into a 200 ml/min constant air stream (Sable Systems intelligent mass flow control unit) and removed via vacuum. A miniature photoionization detector (miniPID, Aurora Scientific) was used to confirm presence and absence of odor during and after odor pulse.

Supplemental Information

Supplemental Information includes Supplemental Experimental Procedures and can be found with this article online at http://dx.doi.org/10.1016/j.cub.2014.12.012.

Author Contributions

S.M.W., J.W.A., and M.A.F. designed experiments. A.G., D.E.K., and C.L. provided reagents and advised on genetic procedures. S.M.W., J.W.A., P.L., J.N., A.L.W., M.F.K., and C.L. collected and/or analyzed data. S.M.W. and M.A.F. wrote the manuscript.

Acknowledgments

Support for this work was provided by the National Institute of Mental Health (R01 MH076900 to D.E.K.), the Brain and Behavior Research Foundation, and the Joanne and George Miller and Family Endowed Chair in Depression Research at the UCLA Brain Research Institute (D.E.K.); the Wellcome Trust (WT085026MA to C.L.); and the Howard Hughes Medical Institute and US Air Force Office of Scientific Research (FA9550-12-1-0034) (M.A.F.).

Received: October 16, 2014
Revised: November 13, 2014
Accepted: December 4, 2014
Published: January 22, 2015

References

1. Shams, L., and Kim, R. (2010). Crossmodal influences on visual perception. Phys. Life Rev. *7*, 269–284.
2. Amedi, A., Malach, R., Hendler, T., Peled, S., and Zohary, E. (2001). Visuo-haptic object-related activation in the ventral visual pathway. Nat. Neurosci. *4*, 324–330.
3. Watkins, S., Shams, L., Josephs, O., and Rees, G. (2007). Activity in human V1 follows multisensory perception. Neuroimage *37*, 572–578.
4. Meredith, M.A., and Stein, B.E. (1986). Visual, auditory, and somatosensory convergence on cells in superior colliculus results in multisensory integration. J. Neurophysiol. *56*, 640–662.
5. Chow, D.M., Theobald, J.C., and Frye, M.A. (2011). An olfactory circuit increases the fidelity of visual behavior. J. Neurosci. *31*, 15035–15047.
6. Duistermars, B.J., and Frye, M.A. (2008). Crossmodal visual input for odor tracking during fly flight. Curr. Biol. *18*, 270–275.
7. Frye, M.A., Tarsitano, M., and Dickinson, M.H. (2003). Odor localization requires visual feedback during free flight in Drosophila melanogaster. J. Exp. Biol. *206*, 843–855.
8. Stewart, F.J., Baker, D.A., and Webb, B. (2010). A model of visual-olfactory integration for odour localisation in free-flying fruit flies. J. Exp. Biol. *213*, 1886–1900.

9. Levy, P., and Larsen, C. (2013). Odd-skipped labels a group of distinct neurons associated with the mushroom body and optic lobe in the adult Drosophila brain. J. Comp. Neurol. *521*, 3716–3740.
10. Krapp, H.G., Hengstenberg, B., and Hengstenberg, R. (1998). Dendritic structure and receptive-field organization of optic flow processing interneurons in the fly. J. Neurophysiol. *79*, 1902–1917.
11. Schnell, B., Joesch, M., Forstner, F., Raghu, S.V., Otsuna, H., Ito, K., Borst, A., and Reiff, D.F. (2010). Processing of horizontal optic flow in three visual interneurons of the Drosophila brain. J. Neurophysiol. *103*, 1646–1657.
12. Suver, M.P., Mamiya, A., and Dickinson, M.H. (2012). Octopamine neurons mediate flight-induced modulation of visual processing in Drosophila. Curr. Biol. *22*, 2294–2302.
13. Grygoruk, A., Chen, A., Martin, C.A., Lawal, H.O., Fei, H., Gutierrez, G., Biedermann, T., Najibi, R., Hadi, R., Chouhan, A.K., et al. (2014). The redistribution of Drosophila vesicular monoamine transporter mutants from synaptic vesicles to large dense-core vesicles impairs amine-dependent behaviors. J. Neurosci. *34*, 6924–6937.
14. Land, M.F. (1973). Head movement of flies during visually guided flight. Nature *243*, 299–300.
15. Schilstra, C., and van Hateren, J.H. (1999). Blowfly flight and optic flow. I. Thorax kinematics and flight dynamics. J. Exp. Biol. *202*, 1481–1490.
16. Schilstra, C., and van Hateren, J.H. (1998). Stabilizing gaze in flying blowflies. Nature *395*, 654.
17. Hengstenberg, R. (1991). Gaze control in the blowfly Calliphora: a multisensory, two-stage integration process. Semin. Neurosci. *3*, 19–29.
18. Duistermars, B.J., Care, R.A., and Frye, M.A. (2012). Binocular interactions underlying the classic optomotor responses of flying flies. Front. Behav. Neurosci. *6*, 6.
19. Götz, K.G. (1968). Flight control in Drosophila by visual perception of motion. Kybernetik *4*, 199–208.
20. Reiser, M.B., and Dickinson, M.H. (2008). A modular display system for insect behavioral neuroscience. J. Neurosci. Methods *167*, 127–139.
21. Theobald, J.C., Ringach, D.L., and Frye, M.A. (2010). Dynamics of optomotor responses in Drosophila to perturbations in optic flow. J. Exp. Biol. *213*, 1366–1375.
22. Haikala, V., Joesch, M., Borst, A., and Mauss, A.S. (2013). Optogenetic control of fly optomotor responses. J. Neurosci. *33*, 13927–13934.
23. Maisak, M.S., Haag, J., Ammer, G., Serbe, E., Meier, M., Leonhardt, A., Schilling, T., Bahl, A., Rubin, G.M., Nern, A., et al. (2013). A directional tuning map of Drosophila elementary motion detectors. Nature *500*, 212–216.
24. Krishnan, P., Duistermars, B.J., and Frye, M.A. (2011). Odor identity influences tracking of temporally patterned plumes in Drosophila. BMC Neurosci. *12*, 62.
25. Chen, T.-W., Wardill, T.J., Sun, Y., Pulver, S.R., Renninger, S.L., Baohan, A., Schreiter, E.R., Kerr, R.A., Orger, M.B., Jayaraman, V., et al. (2013). Ultrasensitive fluorescent proteins for imaging neuronal activity. Nature *499*, 295–300.
26. Tuthill, J.C., Chiappe, M.E., and Reiser, M.B. (2011). Neural correlates of illusory motion perception in Drosophila. Proc. Natl. Acad. Sci. USA *108*, 9685–9690.
27. Chiappe, M.E., Seelig, J.D., Reiser, M.B., and Jayaraman, V. (2010). Walking modulates speed sensitivity in Drosophila motion vision. Curr. Biol. *20*, 1470–1475.
28. Jung, S.N., Borst, A., and Haag, J. (2011). Flight activity alters velocity tuning of fly motion-sensitive neurons. J. Neurosci. *31*, 9231–9237.
29. van Breugel, F., Suver, M.P., and Dickinson, M.H. (2014). Octopaminergic modulation of the visual flight speed regulator of Drosophila. J. Exp. Biol. *217*, 1737–1744.
30. Longden, K.D., and Krapp, H.G. (2010). Octopaminergic modulation of temporal frequency coding in an identified optic flow-processing interneuron. Front. Syst. Neurosci. *4*, 153.
31. de Haan, R., Lee, Y.-J., and Nordström, K. (2012). Octopaminergic modulation of contrast sensitivity. Front Integr Neurosci *6*, 55.
32. Feinberg, E.H., Vanhoven, M.K., Bendesky, A., Wang, G., Fetter, R.D., Shen, K., and Bargmann, C.I. (2008). GFP Reconstitution Across Synaptic Partners (GRASP) defines cell contacts and synapses in living nervous systems. Neuron *57*, 353–363.
33. Maimon, G., Straw, A.D., and Dickinson, M.H. (2010). Active flight increases the gain of visual motion processing in Drosophila. Nat. Neurosci. *13*, 393–399.
34. Farooqui, T. (2012). Review of octopamine in insect nervous systems. Open Access Insect Physiol. *4*, 1–17.
35. van Breugel, F., and Dickinson, M.H. (2014). Plume-tracking behavior of flying Drosophila emerges from a set of distinct sensory-motor reflexes. Curr. Biol. *24*, 274–286.
36. Burke, C.J., Huetteroth, W., Owald, D., Perisse, E., Krashes, M.J., Das, G., Gohl, D., Silies, M., Certel, S., and Waddell, S. (2012). Layered reward signalling through octopamine and dopamine in Drosophila. Nature *492*, 433–437.
37. Gordon, M.D., and Scott, K. (2009). Motor control in a Drosophila taste circuit. Neuron *61*, 373–384.
38. Maimon, G., Straw, A.D., and Dickinson, M.H. (2008). A simple vision-based algorithm for decision making in flying Drosophila. Curr. Biol. *18*, 464–470.
39. Fox, J.L., Aptekar, J.W., Zolotova, N.M., Shoemaker, P.A., and Frye, M.A. (2014). Figure-ground discrimination behavior in Drosophila. I. Spatial organization of wing-steering responses. J. Exp. Biol. *217*, 558–569.
40. Akerboom, J., Chen, T.-W., Wardill, T.J., Tian, L., Marvin, J.S., Mutlu, S., Calderón, N.C., Esposti, F., Borghuis, B.G., Sun, X.R., et al. (2012). Optimization of a GCaMP calcium indicator for neural activity imaging. J. Neurosci. *32*, 13819–13840.

Current Biology

Supplemental Information

Olfactory Neuromodulation of Motion Vision Circuitry in *Drosophila*

Sara M. Wasserman, Jacob W. Aptekar, Patrick Lu, Jade Nguyen, Austin L. Wang, Mehmet F. Keles, Anna Grygoruk, David E. Krantz, Camilla Larsen, and Mark A. Frye

Supplemental Experimental Procedures

Behavior

Closed Loop Magnetic Tether Flight Simulator

A 30 deg wide vertical stripe was rotated around the arena for 60 s at the start of each experiment to verify that an individual animal was able to appropriately orient at all points around the arena. This same stripe was oscillated at 90 deg to visually attract the flies to the odor nozzle. A static wide-field pattern (30 deg spatial wavelength, 94% pattern contrast, 78 cd/ m^2) was presented for the duration of each experiment. Odor was delivered at the location marked as 180-deg and odor stimuli included apple cider vinegar (Ralphs Brand) or water. Individual flies were not run more than three times through a single experiment and animals that stopped flying more than three times were discarded. Flies included in the analyses were required to have acquired the odor plume for at least one frame (30 frames/sec) within the first five seconds. Analyses were performed using custom-written MATLAB software as previously described [S4,S5].

Rigid Tether Flight Simulator

Each experiment began with a period of closed-loop bar fixation wherein the fly is able to actively control the position of the bar[S6]. Flies that were not able to stabilize the bar at the start of an experiment were discarded. Flies were presented with 8 seconds of open-loop ground motion from one of 8 randomly selected spatial stimulus patterns. Half of the trials were randomly paired with an apple cider vinegar plume and half with a water plume. Each test trial was interspersed with periods of closed-loop bar tracking.

Calcium Imaging

Once anesthetized, flies were wedged into a 1/32 inch slit cut into 0.001 inch thick stainless steel shim (304 Stainless Shim. Trinity Brand Industries. Countryside, IL) in a custom-built acetal stage. The cervical connective was flexed downwards to bring the posterior surface of the head capsule flush with the shim, and the rim of the cuticle was fixed in place with dental acrylic[S7]. The proboscis was fixed to prevent motion. The posterior cuticle was cut out and removed with a pair of sharpened Dumont #5 forceps (Fine Science Tools. Foster City, CA). Overlying tissues were removed to provide clear access to the optic lobe. During imaging, a perfusing solution was flowed through the well at a rate of ~1ml/min via a gravity drip regulated by an in-line valve. Perfusate passed through an in-line temperature regulator (Warner Instruments. Hamden, CT) at 19 degrees C, resulting in well temperature of 21-23 degrees C throughout the experiment. Perfusate saline is based on [S8].

Visual Stimuli for Odd tuning experiments

To characterize the subset of the ODD cell group with dendrites in the lobula plate, we performed both directional and spatiotemporal frequency-tuning experiments. In each case, we used square-wave gratings at 100% contrast. For the directional tuning, animals were shown a square-wave grating with a 27 deg spatial wavelength that drifted at a velocity of 27

deg/s for 5 seconds. For each trial, the grating was oriented along one of 36 evenly spaced directions spanning the circle. Each experiment consisted of a single repetition of these 36 trials, presented in random order, interleaved with 2 second rest periods. For spatiotemporal frequency tuning, animals were presented with a square wave grating with a spatial period of 27 deg was drifted in the preferred direction (back-to-front) at temporal frequencies indicated in Figure 2H.

Image Processing

Acquired movies were aligned in two dimensions to a reference image (typically the first frame of the movie) with a built-in two-dimensional correlation algorithm (Slidebook, Intelligent Imaging Innovations, Inc. Denver, CO). An image of the mean fluorescence intensity over time was then exported to MatLab where a customized algorithm was used to parcelate the image into isoluminant regions of interest (ROIs). All data was downsampled to 10Hz. ΔF/F values were calculated by dividing by the mean fluorescence over frames 30 to 70 (3-7s). Mean maximum ΔF/F values were identified within 20 frames on either side of the maximum response during the stimulus period.

HSE , Hx and T4T5 imaging

A single identifiable ROI was selected for Hx and HSE imaging with GCaMP6m. [S9, S10] ROIs for T4T5 were manually assigned to three groups according to the dendritic or axonal locations within the medulla, lobula, or lobula plate. Responses from all three regions did not show significant difference from each other. Therefore, we display only responses within the lobula plate as a representative (Figure 3). Tdc2-Gal4 processing utilized GCaMP6s [S9].

Semi-automatic noise removal for T4T5 and Tdc2

Unlike the tangential cells Hx and HSE, T4T5 and Tdc2 cells are densely tiled within the lobula plate, thus it was necessary to measure their response to stimuli as a population, rather than from a single, repeatable ROI. We began by tiling the movie-recording with a mask that subdivided each recording into equiluminant channels. To remove background ROIs and noise, we developed a recursive principal component analysis approach. To avoid privileging bright ROIs in our analysis, we subtracted the mean value from each row of the matrix and normalized by the standard deviation of luminance over the movie. We then treated each sample as an independent component and each ROI as an independent measurement of that component. We next performed Principle Components Analysis (PCA) and used k-means clustering to group ROIs based off similarity of coefficients across the first four principal components. We identified outlying clusters of points and excluded or included these clusters from further analysis if the non-normalized, non-mean subtracted was low. These ROIs generally corresponded to the background or non-responsive parts of the image. ROIs that responded in a systematically different manner from another subset were separated for the next level of analysis. Once we excluded background ROIs from further analysis, we then performed the same analysis on the remaining ROIs and again looked for systematic differences by visualizing the projections of the remaining ROIs into a new space of principal components. Once we no longer found systematic sources of variation, the process

terminated. As reported in this study, this technique requires user interaction, although it could be automated.

Tdc2 ROI Pruning

Because the Tdc2 cellular processes are diffuse and small in caliber within the lobula plate, we found these recordings to be highly sensitive to animal motion. For this reason, before performing the recursive principle components analysis to remove noise pixels and background, we first excluded all animals where the recordings showed a large amount of motion artifacts that could not be resolved by automatic image alignment. The reported results are from N=6 animals.

Confocal Imaging

Images shown in Figures 2B, 3D, 3G, 4C were obtained via the following protocol. 2-5 days old female flies were dissected in 1x PBS and fixed in 4% paraformaldehyde followed by 3X15 minutes washes in 1x PBS/ 0.3% Triton X-100 (PBST). Brains were then blocked in 5% goat serum diluted in 0.3% PBST for 30 min and incubated in primary antibodies 2 days at (4 °C). Following 3x15 minutes washes in PBST, brains were incubated in secondary antibodies 2 days at (4 °C). Lastly, brains were mounted in Vectashield (Vector Laboratories) on a microscope slide. 2 μm stacks were taken using a Zeiss 710 confocal microscope and images were analyzed with ImageJ (National Institutes of Health). The following primary and secondary antibodies were used: mouse anti-nc82 (1:10, Developmental Studies Hybridoma Bank, University of Iowa, http://dshb.biology.uiowa.edu/bruchpilot), rabbit anti-GFP (1:1000, Molecular Probes, A11122), goat anti-rabbit Alexa 488 (1:200, Molecular Probes, A11034) and goat anti-mouse Alexa 568 (1:200, Molecular Probes, A11031)

GFP reconstitution across synaptic partners (GRASP)

Adult brains were dissected in cold PBS and fixed in a 4% paraformaldehyde/PBS solution for 30 min at room temperature. Brains were then washed several times in PBS containing 0.5% Triton X-100 (PBT) followed by a 2 hour incubation in 1% goat serum (GS) before antigen detection with primary antibody overnight at 4 C° in PBT + 0.5% GS. Following primary antibody incubation brains were washed for 8 hours and incubated overnight in secondary antibody at 4 C° followed by washing for 1 day. Brains were mounted in PBS and viewed using a Zeiss 510 confocal microscope using an air x20. The following antibodies were used: mouse monoclonal anti-Bruchpilot (nc82) (Developmental Studies Hybridoma Bank) (1:10 dilution) and polyclonal rabbit anti-GFP (Invitrogen) (1:400 dilution). Secondary antibodies (Invitrogen) were: Alexa Fluor 488 donkey anti-rabbit, and Alexa Fluor 546 goat-anti-mouse. These were used at a 1:500 dilution.

Supplemental references

S1. Levy, P., and Larsen, C. (2013). Odd-skipped labels a group of distinct neurons associated with the mushroom body and optic lobe in the adult Drosophila brain. J. Comp. Neurol. *521*, 3716–3740.

S2. Burke, C. J., Huetteroth, W., Owald, D., Perisse, E., Krashes, M. J., Das, G., Gohl, D., Silies, M., Certel, S., and Waddell, S. (2012). Layered reward signalling through octopamine and dopamine in Drosophila. Nature *492*, 433–437.
S3. Gordon, M. D., and Scott, K. (2009). Motor control in a Drosophila taste circuit. Neuron *61*, 373–384.
S4. Duistermars, B. J., and Frye, M. A. (2008). Crossmodal visual input for odor tracking during fly flight. Curr. Biol. *18*, 270–275.
S5. Maimon, G., Straw, A. D., and Dickinson, M. H. (2008). A simple vision-based algorithm for decision making in flying Drosophila. Curr. Biol. *18*, 464–470.
S6. Chow, D. M., Theobald, J. C., and Frye, M. A. (2011). An olfactory circuit increases the fidelity of visual behavior. J. Neurosci. *31*, 15035–15047.
S7. Seelig, J. D., Chiappe, M. E., Lott, G. K., Dutta, A., Osborne, J. E., Reiser, M. B., and Jayaraman, V. (2010). Two-photon calcium imaging from head-fixed Drosophila during optomotor walking behavior. Nat. Methods *7*, 535–540.
S8. Wilson, R. I., Turner, G. C., and Laurent, G. (2004). Transformation of olfactory representations in the Drosophila antennal lobe. Science *303*, 366–370.
S9. Chen, T.-W., Wardill, T. J., Sun, Y., Pulver, S. R., Renninger, S. L., Baohan, A., Schreiter, E. R., Kerr, R. a, Orger, M. B., Jayaraman, V., et al. (2013). Ultrasensitive fluorescent proteins for imaging neuronal activity. Nature *499*, 295–300.
S10. Akerboom, J., Chen, T.-W., Wardill, T. J., Tian, L., Marvin, J. S., Mutlu, S., Calderon, N. C., Esposti, F., Borghuis, B. G., Sun, X. R., et al. (2012). Optimization of a GCaMP calcium indicator for neural activity imaging. J. Neurosci. *32*, 13819–13840.
